# Mutagenesis of the *rpoS* gene involved in alteration of outer membrane composition

**Published:** 2019-02

**Authors:** Sayyed Shahryar Rahpeyma, Jamshid Raheb

**Affiliations:** Department of Molecular Medicine, National Institute of Genetic Engineering and Biotechnology (NIGEB), Tehran, Iran

**Keywords:** *rpoS*, *Flexibacter chinesis*, Transposon, Suicide vector, Transmission electron microscopy

## Abstract

**Background and Objectives:**

*rpoS* is a bacterial sigma factor of RNA polymerase which is involved in the expression of the genes which control regulons and play a critical role in survival against stresses. Few suitable vectors are available which could be maintained successfully in *Flexibacter chinesis* cells and could in particular be used as a suicide vector to make mutation in the *rpoS* gene. The aim of this study was to investigate if *rpoS* mutagenesis has impact on bacterial morphology in addition to cell division.

**Materials and Methods:**

A 0.603 kb BamHI-PstI fragment subclone of pICRPOS38Ω was cloned into linearized pLYLO3. The final construct, pLRPOS38 suicide vector, was introduced into *Flexibacter chinesis*. Then the cytoplasm of mutant strain and wild-type were investigated by transmission electron microscopy.

**Results:**

After successful subcloning of suicide vector into *F. chinesis*, based on TEM study, it was demonstrated that mutation in *rpoS* gene leads to decomposition of outer membrane of *F. chinesis*.

**Conclusion:**

A suitable vector to make suicide mutation in *rpoS* was constructed for *F. chinesi*.

## INTRODUCTION

*rpoS* controls a large regulon (1, 2) and plays a critical role in cell survival in stress conditions such as near-UV exposure (3), thermal stress (4), prolonged starvation (5), oxidative stress (3) and low pH (6, 7). Also *rpoS* is important for genome instability (8), switching changes normally from error-free double-strand break repair into an error-prone process under stress (9), phase variation (8), mutagenic processes in growth-limiting environments of bacteria (10, 11) and spontaneous mutations (12). In some cases which several promoters involved in regulation of genes, only one gene is under control of δ^S^ (13, 14).

The *rpoS* gene is located in a structural gene with one operon common with *nlpD* encoding a lipoprotein. There is two promoters not regulated by growth phase and in experiential growth phase demonstrated a low level of *rpoS* expression (5). The *rpoS* transcription is controlled by the cAMP-CRP complex negatively. There is a transcriptional start site for *rpoS* gene, however there is no report of involvement of this site in *rpoS* transcriptional control (15, 16). Every attempt to manipulate a suitable construct, using the conventional cassettes and suicide vectors, which usually are used with *E. coli* and other Gram-negative bacteria failed.

In this study a new technique was reported which proved suitability for the genetic manipulation of gilding bacteria (17). In this study, the 5.97 kb pLYLO3 suicide vector was digested and a 0.603 kb Bam HI fragment subclone of pICRPOS38Ω was cloned into linearized pLYLO3. This construct was named pLRPOS38 and introduced into *Flexibacter chinesis* using conjugation method. Then the cytoplasm of mutant strain and wild-type were investigated by transmission electron microscopy.

## MATERIALS AND METHODS

### Bacterial growth media and conditions.

All bacterial strains were grown in Luria Agar (5 g/l NaCl, 15 g/l Agar, 10 g/l tryptone, 5 g/l yeast extract) or on Luria Broth (5 g/lNaCl, pH 7.2, 5 g/l yeast extract, 10 g/l Bacto tryptone).

### Amplification of the *rpoS* gene.

Primers which were as follow: 5′GGGGGATCCCGTCAAGGGAT-CACGGGTAGGAGCCAC3′ (forward), and 5′GG-GGAATTCTTCAACCTGAATCTGGCGAACAC-GTTC3′ (Reverse) were used to amplify the *rpoS* gene in PCR assay. Amplification was carried out using High Fidelity PCR Master Kit (Roche) and a Perkin-Elmer (USA) DNA thermal cycler. Amplification conditions were 94°C for 45 sec (1 cycle), 94°C for 1 min, 65°C for 45 sec, 72°C for 30 sec (30 cycles). The PCR products were purified using High Pure Product Purification Kit (Roche).

### Conjugation method.

The method described by Maniatis et al. 1989, was used with slight modification. *E. coli* S17-1 was used as the donor for the transfer of the recombinant plasmid, pLRPOS38 into *F. chinensis*. The plasmid RP4 is an IncP type plasmid which is integrated into the chromosome of *E. coli* S17-1 and the plasmid pLYLO3, contains an *oriT* (transfer origin) from PK2, an incP1 plasmid, which is recognized by IncPα plasmids, such as RP4, but not by Incpβ plasmids. The recombinant plasmid, pLRPOS38, was transferred into the *E. coli* S17-1 by transformation and the recombinant *E. coli* S17-1 was used as donor to introduce the recombinant plasmid, pLRPOS38 into *F. chinensis* as recipient, using bi-parental conjugation method. Then the trans-conjugated bacteria were cultivated onto LB agar containing 200 μg erythromycin to select for trans-conjugants. The plates were incubated for 2 to 3 days at 30°C (18, 19).

### Transmission electron microscopy.

Samples were prepared and examined using the Jeol JEM-100S transmission electron microscope with an 80 kV accelerating voltage. Photographs were taken using Kodak Panasonic film, which was developed in Kodak D-19 developer at 20°C for 3 min and fixed in Kodak fixer.

## RESULTS

### Strategy for mutagenesis in the *rpoS* gene of *F. chinesis*.

**Fig. 1.**
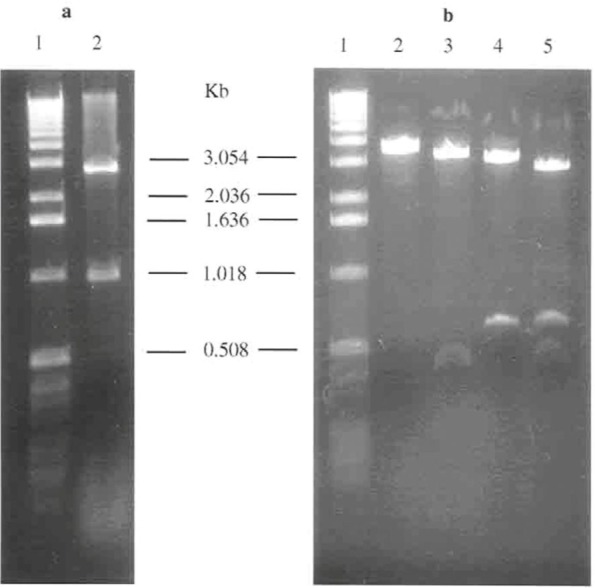
a) Miniprep of pICRPOS38 was digested with a number of restriction enzymes. Lane 1 is λ ladder of 1 kb DNA as a molecular size. Lane 2 is pICRPOS38 digested with EcoRI and BamHI b) Restriction digestion of pICRPOS38 at a unique PmlI site. Lane 1 is λ ladder of 1 kb DNA as a molecular size. Lane 2 is pICRPOS38 digested with PmlI. Lane 3 is pICRPOS38 digested with PmlI and EcoRI. Lane 4 is pICRPOS38 digested with PmlI and BamHI. Lane 5 is pICRPOS38 digested with PmlI, EcoRI and BamHI.

The strategy for the mutation of the *rpoS* gene was the interruption of the gene by the insertion of an interposon (Ω) into the gene. A one kb EcoRI/BamHI fragment of the *rpoS* gene subclone of pBRPOS38 was cloned into pIC19H which provided some convenient poly cloning site for the construction. The construct was named pICRPOS38. The restriction map of the miniprep of the *rpoS* gene in this construct is shown in Fig. 1a. A unique Pml I site was approximately in the middle of the gene and unique for the cloning of pICRPOS38. The restriction digestion of Pml I site approximately located in the middle of the *rpoS* gene is shown in Fig. 1b. This site was used for insertion of a 0.8 kbp interposon into the *rpoS* gene.

### Interruption of the *rpoS* gene of *F. chinesis*

**Fig. 2.**
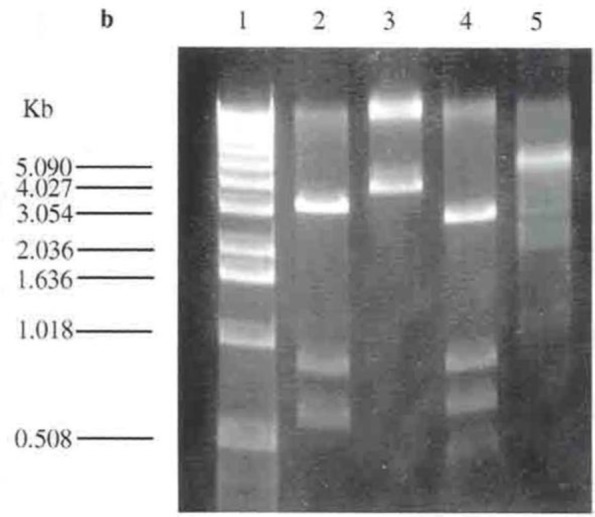
Restriction digestion of pICRPOS38Ω. Lane 1 is λ ladder of 1 kb DNA as a molecular size. Lane 2 is pICRPOS38Ω digested with PstI. Lane 3 is undigested pICRPOS38Ω as a control. Lane 4 is pICRPOS38Ω digested with HindII and Lane 5 is pICRPOS38Ω digested with BamHI.

A 0.8 kb Ω fragment consisting of the CM (chloramphenicol) resistance gene of the pKRB10 plasmid was digested with Hinc II (which has recognition sites at both ends of the cassette) and purified. The constructed pICRPOS38 was linearized by digestion at the unique Pml I site. Both the linearized pI-CRPOS38 and Ω fragments were blunt ended through the digestion with Hinc II and Pml I. The Ω fragment was ligated into the Pml I site of pICRPOS38 yielding pKRPOS38Ω. This was transformed into *E. coli* TG1 and selection for CM resistance was made which confirmed that the Ω fragment had been inserted into the *rpoS* gene. pKRPOS38Ω was digested with BamHI with four sites. A nondigested sample was used as the control. In Fig. 2 the 0.8 kb bands in lanes 2 and 4 belong to the Ω fragment.

### Subcloning of the *rpoS* mutation into the suicide vector, pKNG101.

**Fig. 3.**
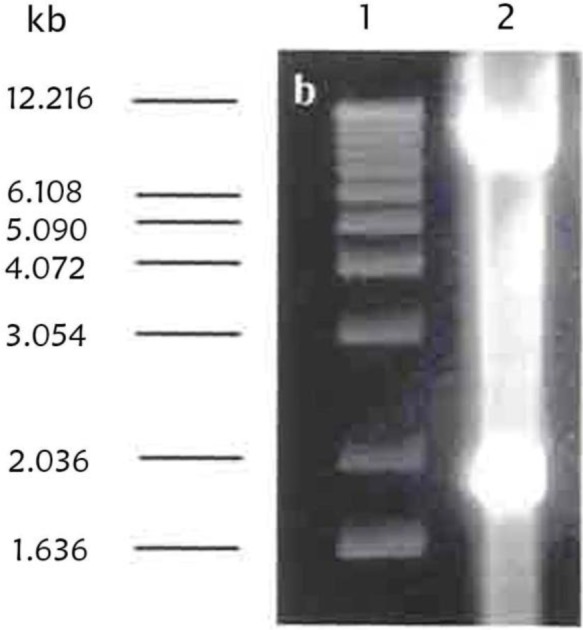
Restriction digestion of pKRPOS38Ω. Lane 1 is λ ladder of 1 kb DNA as a molecular size. Lane 2 is pKRPOS38Ω digested with SalI and BamHI.

The mutated *rpoS* gene with the interposon digested from pICRPOS38Ω at the BglII and BamHI sites was introduced to a linearized pKNG101 digested at a BamHI and dephosphorylated to prevent self-ligation. The linearized vector and *rpoS* mutant gene were ligated and the construct was named pKRPOS38Ω. Restriction analysis was carried out to confirm the presence of a 1.8 kb fragment of the mutated *rpoS* gene in pKRPOS38Ω. As the BglII and BamHI sites had the same ends, they would ligate but neither BamHI nor BglII recognized the BamHI and BglII ligated site. pKRPOS38Ω was digested using four enzymes in two separate digestions. At first, digestion was carried out with SalI and BamHI. Due to the orientation of the insertion in the vector, two possibilities could occur with this digestion. Either the BglII downstream of the mutated *rpoS* gene could be ligated into the BamHI site located next to the SalI site in the vector, subsequent digestion of SalI and BamHI miniprep would result in a 1.8 kb insertion and 6.8 kb vector band; or the BglII end could be ligated to the BamHI site downstream of the *rpoS* mutated gene located next to Bst-BI of the vector, then digestion at the SalI and BamHI sites would not result in any bands. The digestion of SalI and BamHI resulted in 1.8 kb fragment which belonged to the mutated *rpoS* gene and 6.8 kb vector fragment which showed that the first possibility had occurred here (Fig. 3).

### Introduction of plasmid pKRPOS38Ω into *F. chinesis* cells.

Initially electroporation was attempted to transfer the DNA into *F. chinesis*. Subsequently, conjugation was employed for the introduction of plasmid by tri-parental mating. Every attempt to introduce pKRPOS38Ω by electroporation was unsuccessful, probably because of the restriction of transferred DNA by the *F. chinesis* restriction system. Conjugation was widely used to transfer pKRPOS38Ω into *F. chinesis* cells by a tri-parental mating method. One of the three parents was *E. coli* GJ342 which contains the helper plasmid, JC2926; the second parent was *E. coli* SM10λpir. The construct can only replicate in this strain because it supplies in Trans form of the π protein encoded by the λpir gene, such as in the *E. coli* strain lysogenized with a λpir transducing λ phage. All the attempts using conjugation to transfer pKRPOS38Ω into the *F. chinesis* were unsuccessful due to unknown factors.

Introduction of some plasmids, such as pBlue+, into *F. chinesis* was attempted alongside *E. coli* ML30 as a control. Electroporation, different methods of transformation and conjugation were used to transfer this plasmid into *F. chinesis*. Although all the methods were highly successful with the control, no *F. chinesis* colonies were found. The positive control proved that the transformation methods were successful, but due to unknown factors the plasmid was not maintained in *F. chinesis*.

### Introduction of Tn4351 into *F. chinesis* on the broad host range IncPβ plasmid R751 (R751::Tn4351).

**Fig. 4.**
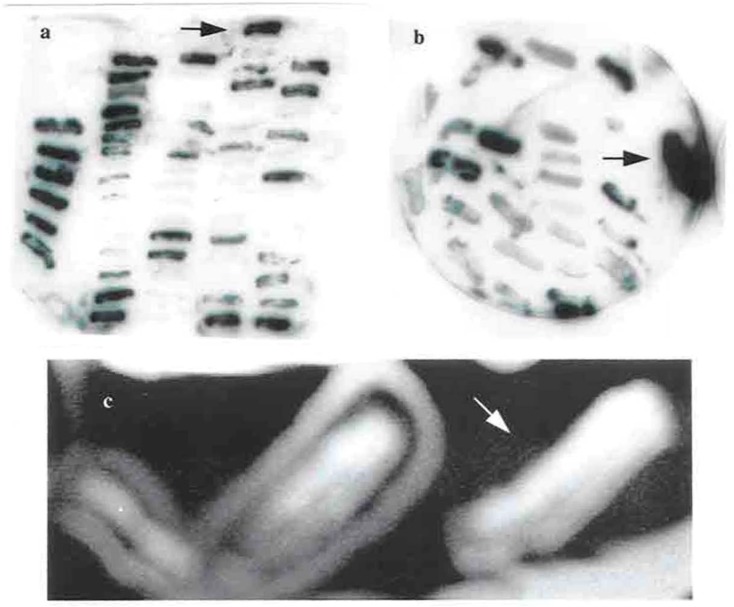
Colony blot hybridization for Tn4351 detection and a none-spreading mutant of *F. chinesis*. a. DNA from erythromycin-resistance colonies was transferred to nylon membranes in duplicate and probed with radiolabeled pVOHI to detect Tn4351. The positive colons are arrowed. b. DNA from erythromycin-resistance colonies was transferred to nylon membranes in duplicate and probed with radiolabeled R751 to detect the delivery vector. The positive controls are arrowed. c. A none-spreading colony is arrowed.

Attempts were made to introduce Tn4351 on the broad-host-ranged Incβ plasmid R751 (R751::Tn4351) into *F. chinesis*. A construct of R751::Tn4351 was selected for introduction into *F. chinesis* to discover if the introduction and insertion of the vector R751 and the transposition of T4351 into the *F. chinesis* chromosome by a tri-parental mating occurred. One parent was *E. coli* GJ342 which carried a helper plasmid, the second parent was *E. coli* HB101 which contained R751::Tn4351 and the third parent was the *F. chinesis* target strain. 189 colonies were isolated on LB agar plates which in passage in fresh media were able to grow in 200 μgml^−1^ erythromycin. The erythromycin resistance gene is carried by Tn4351. Erythromycin resistance colonies were transfer to LB agar containing 200 μgml^−1^ trimethoprim. None of the colonies could grow in this medium and no free vector (R751) was obtained in plasmid miniprep. This indicates that no replication of R751 occurred. Colony blot hybridization was done separately to discover if Tn4351 and/or R751 had inserted into the chromosome of *F. chinesis*. Duplicated blots were probed separately with radio-labeled pVOHI (for the detection of the Tn4351) and R751 (for the detection of the transposon delivery vector). pVOHI is a derivated of pBR328 that carries Tn4351 but there is no common sequence with R751. Approximately half of the colonies selected from the first screen were positive in the second screen for the detection of Tn4351 (Fig. 4.a). A few colonies were positive for the detection of R751 (Fig. 4.b). A few mutant defective in spreading were isolated (Fig. 4.c) and some auxotroph mutants were also isolated.

### Insertional mutagenesis in *F. chinesis* using the novel pUC19-based suicide vector (pLYO3).

**Fig. 5.**
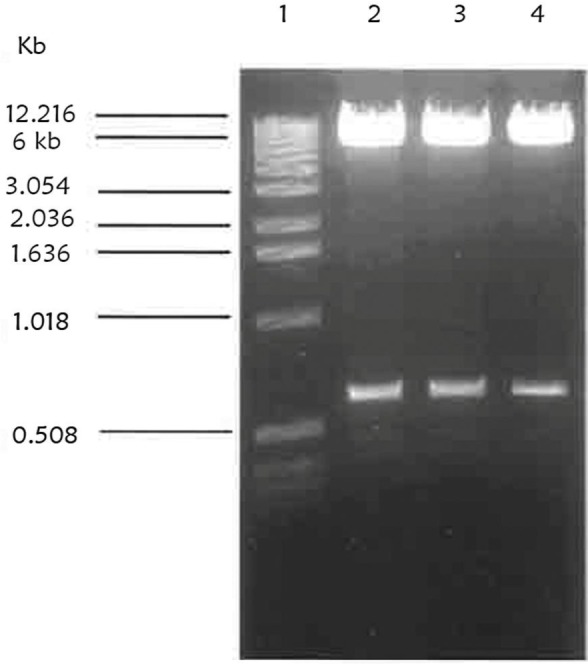
Restriction digestion of construct pLRPOS38. A miniprep of pICRPOS38 was digested. Lane 1 is λ ladder of 1 kb DNA as a molecular size. Lane 2, 3 and 4 show pLRPOS38 digested with BamHI and PstI sites resulting in a0.6 kb partial fragment of *rpoS* gene and 6 kb linearized pLYLO3 vector.

After successfully cloning Tn4351 into *F. chinesis* and isolation of some auxotroph and none spreading mutants, a convenient suicide vector was chosen for disruption mutagenesis in *F. chinesis*. The identification of the *ermF* gene which was carried by Tn4351 and conferred erythromycin resistance in *F. chinesis* was an efficient method for showing that conjugation from *E. coli* to *F. chinesis* had been taken place. pLYLO3 and pUC19 based suicide vector for *Bacteroides* spp., contained *ermF* and also an origin of transfer which would allow conjugative transfer from *E. coli*. The *ermF* gene was not expressed in *E. coli* (20). pLYO3 was constructed originally from pFD160, which contains pUC19 and the cryptic bacteroides plasmid pBI143 and was the basis for the GUC vector. pBI143 also contained a mobilization region that was recognized by the IncP plasmid R751. The region encoding the GUS on a 1.9 kb fragment was removed from pBT101 and cloned into pFD160 which had been digested with BamHI and SstI and named pMJF-1. pMJF-2 was constructed by inserting a 2.9 kb ClaI fragment containing the *ermF* gene from the bacteroides transposon Tn4351 into pMJF1 (the 2.9 kb fragment was digested from a shuttle vector pVJR-1). The pBI143 segment from pMJF-2 was deleted and replaced with an *oriT* region from RK2. *oriT* was recognized by IncPα plasmid, such as RP4, but not by IncPβ plasmids, such as R751. This construct was named pCQW1 (21). Finally a 1.9 kb SmaI-SstI of the region encoding GUS was deleted from pCQW1 and the pCQW1 lacking the SmaI-sstI site was named pLYO3 (22, 23). Therefore, the 5.9 kb pLYO3 suicide vector was digested with BamHI and PstI sites and a 0.603 kb BamHI-PstI fragment subclone of pICRPOS38Ω was cloned into the linearized pLYLO3 digested with BamHI-PstI. Fig. 5 shows the results of the digestion of the 0.603 kb BamHI-PstI fragment from the construct, pLRPOS38.

### Introduction of the construct pLRpOS38 into *F. chinesis* cells by conjugation.

The construct, pLRPOS38 which contained *oriT* to allow conjugation was transferred into *F. chinesis* by a bi-parental conjugation method that one of the parents was *E. coli* S17-1 which contained a derivative of RP4 (RP4-2::Mu-Km::Tn7Tc^r^) integrated into the chromosome (24, 25). The other parent was *F. chinesis* as a recipient strain. The construct containing *oriT* carried the specific mob site which was recognized by IncPα plasmids, such as RP4, and facilitates transfer of the construct from *E. coli* S17-1. It also contained an ampicillin resistance gene which was expressed in *E. coli* and *F. chinesis* and an erythromycin resistance gene which was not expressed in *E. coli* but was expressed in *F. chinesis*. Therefore as the *ermF* gene did not confer erythromycin resistance to *E. coli* strain, the transformed construct in *E. coli* S17-1 was selected with ampicillin. After conjugation had taken place, as the *ermF* gene was not expressed in *E. coli*, the ex-conjugant cells were selected with erythromycin. 26 colonies were isolated on an erythromycin medium. This result demonstrated for the first time that pLYLO3 worked successfully as a suicide vector in gliding bacteria. The *rpoS*::pLYLO3 ex-conjugants were transferred into fresh erythromycin (200 μgml^−1^) medium to eliminate possible pseudo ex-conjugants.

### Selection of recombinant *F. chinesis*.

24 erythromycin resistance colonies are single recombinants since the target gene was disrupted at one site. These cells would also been ampicillin resistant. The recombinant clone was *F. chinesis* carrying pLRPOS38 plasmid. Also the accuracy of cloning was confirmed by colony blot analysis (Fig. 4.a and 4.b).

### Study of cell morphology using transmission electron microscopy (TEM).

The recombinant *F. chinesis* cells had a quite different shape to the starved wild-type cells and no shrinkage in the cytoplasm was observed (Data not shown). TEM study showed that the mutant strain had no outer membrane. This result suggested that the *rpoS* gene was important for composition of outer membrane in *F. chinesis* (Fig. 4.c).

## DISCUSSION

The genetic studies of gliding bacteria had been limited with the exception of studies on one species, *Myxococcus xanthus* (26). Mutant of *M. xanthus* which were defective in motility had been isolated and a number of genes identified which were necessary for colony spreading (17). Following the development of technique for the genetic manipulation of *Bacteroides* spp., a number of techniques were developed to genetically manipulate gliding bacteria (17, 27).

Tn4351 was originally isolated from *Bacteroides fragilis* (28). The transposon was successfully introduced into *Cytophaga succinicans, Flavobacterium meningosepticum, Flexibacter canadiansis, Flexibacter* strain SFI and *Sporocytophaga myxococcoides* by conjugation (17). Tn4351 carried two antibiotic resistance genes. One of the genes coded for resistance to erythromycin and clindamycin was expressed in *Bactroides* but not in *E. coli.* The other gene coded resistance in tetracycline and was expressed in aerobically grown *E. coli*, but not in anaerobically grown *E. coli* or in *Bacteroides.* The transposon of Tn4351 was originally detected in *E. coli* which carried an unstable chimeric plasmid, pSS-2. The mobilization of pSS-2 from one strain of *E. coli* to another leaded to insertion of Tn4351 into R751 (R751::Tn4351) and less frequently to the insertion of Tn4351 into the chromosome. R751 also mobilized pE5-2 which contains a 3.8 kb EcoRI fragment of Tn4351 and can mobilize a cryptic bacteroides plasmid into *Bacteroides* spp. R751 was never detected in any of the *Bacteroides* trans-conjugants that carried pE5-2. This result demonstrates that R751 was not maintained in *Bacteroides*. In addition, there is some evidence that when R751 is inserted in to the bacteroides chromosome, it cannot mobilize out of the *Bacteroides*. Consequently it could be used as a suitable suicide vector in *Bacteroides* species.

Few suitable vectors are available which could be maintained successfully in *F. chinesis* cells and could, in particular, be used as a suicide vector to make mutation in the *rpoS* gene. In this study we used a novel method for mutagenesis of the *rpoS* gene in *F. chinesis.* Initially pKNG101 and pKRPOS38 plasmids were used to mutate the *rpoS* gene in the *F. chinesis* but the attempts were unsuccessful. Then In the bacteroides transposon, Tn4351 was cloned into R751 and transformed into *F. chinesis* successfully. In this method the 5.97 kb pLYLO3 suicide vector was digested and a 0.603 kb BamHI fragment subclone of pICRPOS38Ω was cloned into linearized pLYLO3. This construct was named pLRPOS38 and introduced into *F. chinesis* using conjugation method. The accuracy of cloning was approved by culturing of recombinant strain onto media containing erythromycin and ampicillin and using southern blot analysis. Electron microscopy of starved wild-type and starved recombinant *F. chinesis* showed that the cytoplasm of starved wild-type cells became clear and shrunk leaving cells which had an odd shape and appearance. In recombinant *F. chinesis* the cells had a quite different shape to the starved wild-type cells and no shrinkage in the cytoplasm was observed, previously in *E. coli* it was reported by Ozkanka (1993) (29) and in *Hydrophila* by Lim (1995) (30).
